# A Phase 1/2 study of teclistamab, a humanized BCMA × CD3 bispecific Ab in Japanese patients with relapsed/refractory MM

**DOI:** 10.1007/s12185-024-03884-z

**Published:** 2024-11-28

**Authors:** Tadao Ishida, Yoshiaki Kuroda, Kosei Matsue, Takuya Komeno, Takuro Ishiguro, Jun Ishikawa, Toshiro Ito, Hiroshi Kosugi, Kazutaka Sunami, Kazuko Nishikawa, Kazuhiro Shibayama, Kensuke Aida, Hiroshi Yamazaki, Mitsuo Inagaki, Hisanori Kobayashi, Shinsuke Iida

**Affiliations:** 1 Department of Hematology, Japanese Red Cross Medical Center, Tokyo, Japan; 2 Department of Hematology, NHO Hiroshimanishi Medical Center, Otake, Japan; 3 Department of Internal Medicine, Kameda Medical Center, Kamogawa, Japan; 4 Department of Hematology, NHO Mito Medical Center, Mito, Japan; 5 Department of Internal Medicine, Niigata Cancer Center Hospital, Niigata, Japan; 6 Department of Hematology, Osaka International Cancer Institute, Osaka, Japan; 7 Department of Hematology, NHO Matsumoto Medical Center, Matsumoto, Japan; 8 Department of Hematology, Ogaki Municipal Hospital, Ogaki, Japan; 9 Department of Hematology, NHO Okayama Medical Center, Okayama, Japan; 10 Research and Development Division, Janssen Pharmaceutical K.K, Tokyo, Japan; 11 Department of Hematology and Oncology, Nagoya City University Graduate School of Medical Sciences, Nagoya, Japan

**Keywords:** B-cell maturation antigen, Bispecific antibody, Japanese, Multiple myeloma, Teclistamab

## Abstract

We characterized the safety and efficacy of the bispecific antibody teclistamab in Japanese patients with relapsed/refractory multiple myeloma (RRMM). Patients were pretreated with a proteasome inhibitor (PI), immunomodulatory drug (IMiD), and anti-CD38 monoclonal antibody (mAb). The primary endpoint was frequency and type of treatment-emergent adverse events (TEAEs) in phase 1, and overall response rate (ORR; ≥ partial response [PR]) in phase 2. In phase 1, 14 patients received once-weekly (QW) subcutaneous teclistamab (0.72 mg/kg [n = 5]; 1.5 mg/kg [n = 5]; 3 mg/kg [n = 4]). No dose-limiting toxicities were observed. As of April 2024, 26 phase-2 patients received the recommended phase-2 dose (QW) (RP2D: 1.5 mg/kg) of teclistamab. Biweekly (Q2W) dosing was allowed after maintaining response for ≥ 6 months. At a median follow-up of 14.32 months, ORR was 76.9% (≥ very good PR: 76.9%; ≥ complete response: 65.4%). Median duration of response, progression-free survival, and overall survival were not reached. Common TEAEs included CRS (grade ≤ 2), neutropenia, and infections. No patient had immune effector cell-associated neurotoxicity syndrome (ICANS) and dose reductions. Teclistamab demonstrated deep and durable responses in Japanese patients with RRMM, consistent with the global pivotal MajesTEC-1 study, supporting the potential for a new standard of care for Japanese RRMM patients.

**Supplementary Information:**

The online version contains supplementary material available at 10.1007/s12185-024-03884-z.

## Introduction

Multiple myeloma (MM) is the third most common hematological malignancy worldwide and accounts for ∼1% of all malignant tumors and 10–15% of hematopoietic neoplasms in Japan [1, 2]. It is often marked by frequent relapses until the patients become refractory [3]. The standard therapy for relapsed/refractory MM (RRMM) includes immunomodulatory drugs (IMiDs), monoclonal antibodies (mAbs), proteasome inhibitors (PIs), cytotoxic drug combinations, and high-dose therapy with autologous stem cell transplant, all of which have demonstrated significant survival benefits versus standard-of-care (SOC) therapies [4]. Despite these advances, most patients eventually relapse or become refractory due to factors like drug-resistant clones, antigen escape, suboptimal T-cell function, or adverse drug reactions. These factors, not fully elucidated, render patients progressively resistant to standard RRMM treatments [5, 6]. The prospective and non-interventional LocoMMotion study reported poor outcomes and rapid disease progression with SOC therapies for RRMM, including corticosteroids, IMiDs, alkylating drugs, and anti-CD38 mAbs [7]. The overall response rate (ORR; 29.8%) was low, and the median progression-free survival (PFS; 4.6 months) and median overall survival (OS; 12.4 months) were short for these patients. These findings overall highlight a need for therapies with innovative modes of action that can overcome resistance and improve depth and durability of response, with an acceptable tolerability profile in heavily pretreated patients with RRMM. Therapies that target the B-cell maturation antigen (BCMA) have been approved for patients with RRMM who have received ≥ 3 prior therapies including IMiDs, PIs, and anti-CD38 antibodies. These therapies have doubled the ORR compared with previous SOC [8, 9], with patients achieving deep and durable responses with a manageable safety profile. These have also demonstrated notable minimal residual disease (MRD) negativity rates and antimyeloma activity, advancing the treatment landscape for MM [10].

Teclistamab, is a Humanized immunoglobulin (Ig) G-4 proline, alanine, alanine (IgG-4 PAA) bispecific antibody, that targets the cluster of differentiation (CD)3 receptor complex on T lymphocytes and BCMA on B lymphocytes, resulting in T-cell activation and subsequent lysis of BCMA positive cells [11]. It is the first approved off-the-shelf T-cell redirecting bispecific antibody by the US Food and Drug Administration (FDA) and European medicines agency (EMA) in 2022 [11] for adults with triple-class-exposed RRMM, who have received ≥ 3 prior lines of therapy, based on results from MajesTEC-1 results [12, 13]. MajesTEC-1 is a pivotal phase 1/2 study where subcutaneous (SC) administration of teclistamab (1.5 mg/kg) in a heavily pretreated global MajesTEC-1 population resulted in an ORR of 63% at a median follow-up of 14.1 months [12]. This ORR remained consistent at 63% in the 30.4 month long-term follow-up [14]. Teclistamab is being investigated as a monotherapy for RRMM patients with 1–3 prior lines of therapy and in combination with standard or novel therapies for newly diagnosed MM and RRMM [15–17]. Herein, we report the findings from the phase-1/2 study of SC teclistamab in heavily pretreated Japanese patients with triple-class-exposed RRMM, characterizing the safety and efficacy of the recommended phase-2 dose (RP2D) in this population.

## Methods

### Study design and treatment

This is an ongoing open-label, single-arm, phase-1/2 study (NCT04696809) conducted at 15 sites in Japan. The study has 2 parts: (1) phase 1: the dose-escalation study, to assess the safety and tolerability of the RP2D of teclistamab identified in the first-in-human global MajesTEC-1 study [12, 18]; (2) phase 2: to evaluate the efficacy and safety of the RP2D in Japanese patients with RRMM. All patients provided written informed consent before study entry. Study was approved by relevant institutional review boards and independent ethics committees and conducted per ICH-GCP guidelines, and the Declaration of Helsinki.

Phase 1 had a screening phase and a treatment phase (SC teclistamab was administered once-weekly [QW] on Days 1, 8, and 15 of a 21-day cycle). The interval between step-up dose(s) (SUD) and the first treatment dose was 2–4 days. Teclistamab was assessed in three dose-escalating cohorts (cohort 1: 0.72 mg/kg QW [SUD: 0.06 and 0.24 mg/kg]; cohort 2: 1.5 mg/kg QW [SUD: 0.06 and 0.3 mg/kg]; cohort 3: 3 mg/kg QW [SUD: 0.06, 0.3, and 1.5 mg/kg]; cohort 4 [not enrolled]: 6 mg/kg QW [SUD: 0.06, 0.3, and 1.5 mg/kg]). Phase 2 had a screening phase, a treatment phase, and a long-term follow-up phase until the end of the study. The RP2D was determined based on MajesTEC-1 study [18] and results of dose-limiting toxicity (DLT) evaluation for cohorts 1 and 2 in phase 1 of this study. During the treatment phase, patients received the SUD schedule of teclistamab at 0.06 or 0.3 mg/kg for 2–4 days followed by the treatment dose of 1.5 mg/kg on Days 1, 8, 15, and 22 of a 28-day cycle. In both phases, the dose interval could be changed from QW to Q2W in patients with a partial response (PR) or better for ≥ 6-months (Supplementary Fig. 1).

### Patients

Eligible Japanese patients (aged ≥ 20 years) had documented diagnosis of MM per International Myeloma Working Group (IMWG) diagnostic criteria; Eastern Cooperative Oncology Group performance status of 0 or 1; serum M-protein level ≥ 1.0 g/dL for phase 1 and ≥ 0.5 g/dL for phase 2 or urine M-protein level ≥ 200 mg/24 h or light chain MM; and received a PI, an IMiD, and an anti-CD38 monoclonal antibody as prior lines of therapy. In phase 2, patients who had received ≥ 3 prior lines of therapy; had documented disease progression during or within 12-months of the most recent anti-myeloma therapy; and had undergone ≥ 1 complete treatment cycle for each prior line of therapy (unless progressive disease was the best response) were enrolled. Key exclusion criteria included prior treatment with any BCMA-targeted therapy, unresolved toxicities from previous anticancer treatments, an allogeneic (within 6-months), or an autologous stem cell transplant (within 12-weeks).

### Endpoints and assessments

The primary safety endpoint of phase 1 was frequency and type of treatment-emergent adverse events (TEAEs) including the incidence of DLTs and the secondary endpoints were ORR (defined as a PR or better according to the IMWG response criteria), pharmacokinetic (PK) parameters, pharmacodynamic (PD) markers, and anti-teclistamab antibodies. The primary efficacy endpoint of phase 2 was ORR and the secondary endpoints included duration of response (DOR), rate of ≥ very good partial response (VGPR), complete response (CR), stringent complete response (sCR), time to response (TTR), PFS, OS, MRD negativity rate, frequency and severity of TEAEs, PK parameters, and anti-teclistamab antibodies.

TEAEs coded by the MedDRA, Version 26.0, including DLTs, were graded per National Cancer Institute Common Terminology Criteria for Adverse Events version 5.0, except for cytokine release syndrome (CRS) and immune effector cell-associated neurotoxicity syndrome (ICANS) which were evaluated according to the American Society of Transplantation and Cellular Therapy (ASTCT) guidelines [19]. Response in phase 1, was assessed by the investigators using 2016 IMWG response criteria, and in phase 2, it was based on computer algorithm assessment based on 2016 IMWG response criteria. MRD testing was done by next-generation sequencing (Adaptive Laboratory). Blood samples and bone marrow aspirate were collected for PK, PD, and immunogenicity analyses at prespecified intervals (refer to Supplemental). Serum samples were analyzed for teclistamab concentrations, cytokine profiles, and anti-teclistamab antibodies using validated assays.

### Statistical analysis

In phase 1 (sample size: ≥ 3 patients in each cohort), the DLT assessment (Supplementary Table 1) was performed applying the Bayesian Optimal Interval design with dose-escalation/de-escalation. In phase 2, a sample size of 24 was determined to provide ~ 85% power to declare an ORR > 20% at one-sided significance level of 0.025, assuming an ORR of ≥ 50%. Safety and efficacy analyses were performed in all treated analysis sets (patients who had ≥ 1 dose of study agent) and the data were descriptively summarized. The ORR and its 2-sided 95% exact confidence interval (CI) were calculated. Kaplan–Meier method was used to estimate DOR, PFS, and OS.

PK data were summarized descriptively for the PK analysis set (patients who had ≥ 1 dose of the study agent and had ≥ 1 evaluable concentration measurement of the study agent). Serum PK parameters were assessed using non-compartmental analysis. PK parameters included maximum plasma concentration (C_max_), the area under the plasma concentration–time curve (AUC), and time to maximum concentration (t_max_). Soluble BCMA (sBCMA) was assessed in the PK analysis set, and immunogenicity was assessed in immunogenicity analysis set (patients who received ≥ 1 dose and had ≥ 1 post-dose immunogenicity sample).

## Results

### Patients

Between February 2021 and September 2023 (clinical cutoff), 14 patients received teclistamab in phase1 (cohort 1: n = 5; cohort 2: n = 5; cohort 3: n = 4). The median patient age was 73.5 years, and the median weight was 49.7 kg; the majority were female (78.6%); 57.1% had high-risk cytogenetics; 14.3% had International Staging System (ISS) stage III disease; 64.3% of patients had Eastern Cooperative Oncology Group (ECOG) performance status 0; and 21.4% had ≥ 1 extramedullary plasmacytomas (EMP) (Table 1). Seven (50%) patients discontinued the study as of the clinical cutoff due to progressive disease (4 [28.6%]) or physician’s decision (3 [21.4%]).

**Table 1:** Demographics and baseline characteristics

Characteristic	Phase 1				Phase 2
	Cohort 1 0.72 mg/kg QW (n = 5)	Cohort 2 1.5 mg/kg QW (n = 5)	Cohort 3 3 mg/kg QW (n = 4)	Total (n = 14)	RP2D 1.5 mg/kg QW (n = 26)
Age, median, years	52.0	74.0	74.5	73.5	67.5
≥ 75 years	1 (20)	2 (40)	2 (50)	5 (35.7)	5 (19.2)
Female, n (%)	3 (60.0)	4 (80.0)	4 (100.0)	11 (78.6)	12 (46.2)
Weight, median (range), kg	58.9 (47.0–70.0)	48.7 (39.1–55.9)	43.8 (37.3–58.0)	49.7 (37.3–70.0)	58.0 (37.5–86.4)
ECOG PS, n (%)					
0	3 (60.0)	3 (60.0)	3 (75.0)	9 (64.3)	16 (61.5)
1	2 (40.0)	2 (40.0)	1 (25.0)	5 (35.7)	10 (38.5)
Cytogenetic risk, n^1^ (%)					
High-risk	0	5 (100.0)	3 (75.0)	8 (57.1)	5 (19.2)
t(4;14)	0	4 (80.0)	1 (25.0)	5 (35.7)	0
del(17p)	0	2 (40.0)	2 (50.0)	4 (28.6)	4 (15.4)
t(14;16)	0	0	0	0	1 (3.8)
ISS stage^a^, n (%)					
I	2 (40.0)	3 (60.0)	3 (75.0)	8 (57.1)	16 (61.5)
II	2 (40.0)	1 (20.0)	1 (25.0)	4 (28.6)	7 (26.9)
III	1 (20.0)	1 (20.0)	0	2 (14.3)	3 (11.5)
Time since diagnosis, median (range), years	5.85 (1.6–6.4)	4.19 (2.8–7.7)	3.94 (2.9–6.2)	4.65 (1.6–7.7)	6.36 (0.9–12.1)
Extramedullary Plasmacytomas^2^, n (%)					
0	4 (80.0)	5 (100.0)	2 (50.0)	11 (78.6)	22 (84.6)
≥ 1	1 (20.0)	0	2 (50.0)	3 (21.4)	4 (15.4)
Number of prior LOT, n (%)					
2	1 (20.0)	1 (20.0)	0	2 (14.3)	0
3	0	0	2 (50.0)	2 (14.3)	8 (30.8)
4	1 (20.0)	0	1 (25.0)	2 (14.3)	5 (19.2)
5	0	1 (20.0)	1 (25.0)	2 (14.3)	3 (11.5)
> 5	3 (60.0)	3 (60.0)	0	6 (42.9)	10 (38.5)
Median (range)	6.0 (2–9)	6.0 (2–15)	3.5 (3–5)	5.0 (2–15)	4.5 (3–12)
Prior exposure status, n (%)					
Triple-class^b^	5 (100.0)	5 (100.0)	4 (100.0)	14 (100.0)	26 (100.0)
Penta-drug^c^	3 (60.0)	4 (80.0)	3 (75.0)	10 (71.4)	15 (57.7)
Refractory status, n (%)					
Triple-class^b^	4 (80.0)	2 (40.0)	3 (75.0)	9 (64.3)	17 (65.4)
Penta-drug^c^	2 (40.0)	1 (20.0)	2 (50.0)	5 (35.7)	6 (23.1)
Refractory to last LOT	5 (100.0)	4 (80.0)	4 (100.0)	13 (92.9)	25 (96.2)

*CD* cluster of differentiation, *ECOG PS* Eastern Cooperative Oncology Group Performance Status, *IMiD* immunomodulatory drug, *ISS* International Staging System, *LOT* line of therapy, *mAb* monoclonal antibody, *PI* proteasome inhibitor, *RP2D* recommended phase-2 dose, *QW* once weekly

^a^ISS staging is derived based on serum β2-microglobulin and albumin

^b^ ≥ 1 PI, ≥ 1 IMiD, and 1 anti-CD38 mAb.^c^ ≥ 2 PIs, ≥ 2 IMiDs, and 1 anti-CD38 mAb

^1^Cutoff value of cytogenetic risk: del 17P (p53) = 14% or greater of abnormal cells detection; T(4;14) = 11% or greater of abnormal cells detection; T(14;16) = 12% or greater of abnormal cells detection

^2^A plasma cell neoplasm of soft tissue without bone marrow involvement or other systemic characteristics of MM

From August 2022 to April 2024, 26 patients received the RP2D of teclistamab in phase 2, median patient age was 67.5 years, and weight was 58.0 kg; 46.2% were female; 19.2% had high-risk cytogenetics; 11.5% had ISS stage III disease, 61.5% patients had ECOG performance status 0, and 15.4% had ≥ 1 EMP (Table 1). Ten (38.5%) patients discontinued the study, due to progressive disease (3 [11.5%]), death, treatment rejection, physician’s decision (2 [7.7%], each), and adverse events (AE; 1 [3.8%]).

### Treatment exposure

In phase 1, the median follow-up was 2.53, 16.30, and 12.76 months and the median treatment duration was 1.61, 15.70, and 12.75 months in cohorts 1, 2, and 3, respectively. In phase 2, the median follow-up was 14.32 months, the median treatment duration was 12.9 months, and the median relative dose intensity was 91.7%.

### Safety

In phase 1, no DLTs occurred across all 3 cohorts. Of the 14 patients in phase 1, 13 (92.9%) patients experienced ≥ 1 TEAE; serious TEAEs were reported in 6 (42.9%) patients (Table 2; Supplementary Table 2). No dose reductions or treatment discontinuations due to TEAEs were reported. Infections occurred in 9 (64.3%; any grade) patients; of these 2 (14.3%) patients had a grade 3/4 infections due to COVID-19 pneumonia and sepsis (1[7.1 each]). All CRS events were of grade 1 and neurotoxic events were of grade 1/2 and resolved at clinical cutoff date (Supplementary Tables 3 & 4). There was no reported grade 5 TEAEs or ICANS.

**Table 2:** Treatment-emergent adverse events summary^a^ (all-treated analysis set)

Event, n (%)	Phase 1^b^						Phase 2^c^	
	Cohort 1 0.72 mg/kg QW (n = 5)		Cohort 2 1.5 mg/kg QW (n = 5)		Cohort 3 3 mg/kg QW (n = 4)		RP2D 1.5 mg/kg QW (n = 26)	
	Any grade	Grade ≥ 3	Any grade	Grade ≥ 3	Any grade	Grade ≥ 3	Any grade	Grade ≥ 3
Any TEAE	4 (80.0)	4 (80.0)	5 (100.0)	5 (100.0)	4 (100.0)	4 (100.0)	26 (100.0)	23 (88.5)
Hematologic								
Neutropenia	2 (40.0)	2 (40.0)	4 (80.0)	4 (80.0)	4 (100.0)	4 (100.0)	19 (73.1)	19 (73.1)
Lymphopenia	2 (40.0)	2 (40.0)	2 (40.0)	2 (40.0)	1 (25.0)	0	8 (30.8)	8 (30.8)
Anemia	1 (20.0)	1 (20.0)	2 (40.0)	1 (20.0)	2 (50.0)	1 (25.0)	7 (26.9)	6 (23.1)
Iron deficiency anemia	0	0	0	0	0	0	3 (11.5)	0
Non-hematologic								
Pyrexia	4 (80.0)	0	3 (60.0)	0	0	0	10 (38.5)	1 (3.8)
Hyperglycemia	2 (40.0)	2 (40.0)	1 (20.0)	0	2 (50.0)	0	0	0
Nausea	2 (40.0)	0	0	0	1 (25.0)	0	4 (15.4)	0
Headache	2 (40.0)	0	0	0	0	0	3 (11.5)	0
Hypogammaglobulinemia	1 (20.0)	0	2 (40.0)	0	0	0	15 (57.7)	1 (3.8)
Malaise	1 (20.0)	0	1 (20.0)	0	0	0	0	0
Constipation	1 (20.0)	0	1 (20.0)	0	0	0	0	0
Diarrhea	1 (20.0)	0	0	0	1 (25.0)	1 (25.0)	3 (11.5)	0
Vomiting	1 (20.0)	0	0	0	1 (25.0)	0	4 (15.4)	0
Nasopharyngitis	1 (20.0)	0	1 (20.0)	0	0	0	8 (30.8)	0
Stomatitis	0	0	1 (20.0)	0	1 (25.0)	0	0	0
Hypokalemia	1 (20.0)	0	0	0	1 (25.0)	0	0	0
Insomnia	1 (20.0)	0	2 (40.0)	0	1 (25.0)	0	2 (7.7)	0
Tinea pedis	0	0	2 (40.0)	0	0	0	0	0
Injection-site erythema	0	0	1 (20.0)	0	1 (25.0)	0	8 (30.8)	0
Injection-site pruritus	0	0	0	0	0	0	3 (11.5)	0
Cytomegalovirus infection	0	0	0	0	0	0	3 (11.5)	0
Peripheral edema	0	0	0	0	0	0	3 (11.5)	0
Dental caries	0	0	0	0	0	0	3 (11.5)	0
Rash	0	0	0	0	0	0	3 (11.5)	0
Hyponatremia	0	0	0	0	0	0	3 (11.5)	3 (11.5)
Cytokine release syndrome^d^	2 (40.0)	0	3 (60.0)	0	3 (75.0)	0	21 (80.8)	0
Neurotoxicities	2 (40.0)	0	1 (20.0)	0	0	0	2 (7.7)	0

*AE* adverse event, *ASTCT* American Society for Transplantation and Cellular Therapy, *RP2D* recommended phase-2 dose, *TEAE* treatment-emergent AE, *QW* once weekly

^a^AEs were reported in ≥ 10% of the patients

^b^Clinical data cutoff date for phase 1 is September 2023

^c^Clinical data cutoff date for phase 2 is April 2024

^d^Cytokine release syndrome was graded per ASTCT criteria

In phase 2, all (26 [100.0%]) patients reported ≥ 1 TEAEs, and 23 (88.5%) patients had grade ≥ 3 TEAEs; serious TEAEs were reported in 13 (50.0%) patients (Supplementary Table 2). TEAEs leading to dose delay or dose interruption were observed in 23 (88.5%) patients. No patient had dose reduction due to TEAEs and only one patient had treatment discontinuation due to grade 3 TEAE of hyponatremia (not related to teclistamab) (Supplementary Table 2). Hematologic events were the most frequently (≥ 10%; any grade or grade ≥ 3) reported TEAEs which included neutropenia (19 [73.1%; any grade]), lymphopenia (8 [30.8%; any grade]), and anemia (7 [26.9%; any grade]) (Table 2). Hypogammaglobulinemia occurred in 22 (84.6%) patients as identified through adverse event reports, laboratory analyses (IgG level, < 500 mg per deciliter), and 21 (80.8%) patients received ≥ 1 immunoglobulin replacement therapy for prophylaxis use at any time during the study. Infections occurred in 20 (76.9%; any grade) patients (Supplementary Table 5) and the most common were nasopharyngitis (8 [30.8%]) and cytomegalovirus infection (3 [11.5%]). Grade 3/4 infections were observed in 5 (19.2%) patients. CRS occurred in 21 (80.8%) patients (any grade), where all events were grade 1 (69.2%) and grade 2 (11.5%). All CRS events resolved without treatment discontinuation or dose reduction (Supplementary Table 3). Pyrexia was the most frequent CRS symptom observed in 20 (76.9%) patients. Most CRS events occurred after SUD 1, SUD 2 (9 [34.6%], each SUD) and first treatment dose (4[15.4%]). Median (range) time to CRS onset was 2 (1.0–5.0) days, median duration was 3 (1.0–24.0) days, and 21 (80.8%) patients received supportive measures to treat CRS including tocilizumab and acetaminophen (17 [65.4%] each), intravenous fluids and steroids (3 [11.5%] each). Neurotoxic events occurred in 2 (7.7%) patients (grade 1 headache and somnolence, 1 [3.8%] patient each). No patient experienced ICANS. One (3.8%) patient reported a TEAE of COVID-19. Two (7.6%) patients experienced grade 5 TEAEs; one patient with a large intestine perforation due to colon cancer, and the other with multi-organ dysfunction syndrome (neither was considered treatment-related per investigator).

### Efficacy

In phase 1, 9 out of 14 (64.3%) patients achieved a response (Supplementary Fig. 2); cohort 1: 2 (20.0%) patients (sCR observed in 2 patients), cohort 2: 4 (80.0%) patients (sCR in 2 patients: CR and VGPR in 1 patient each), cohort 3: 3 (75.0%) patients (sCR in 2 patients; VGPR in 1 patient).

**Fig. 1:**
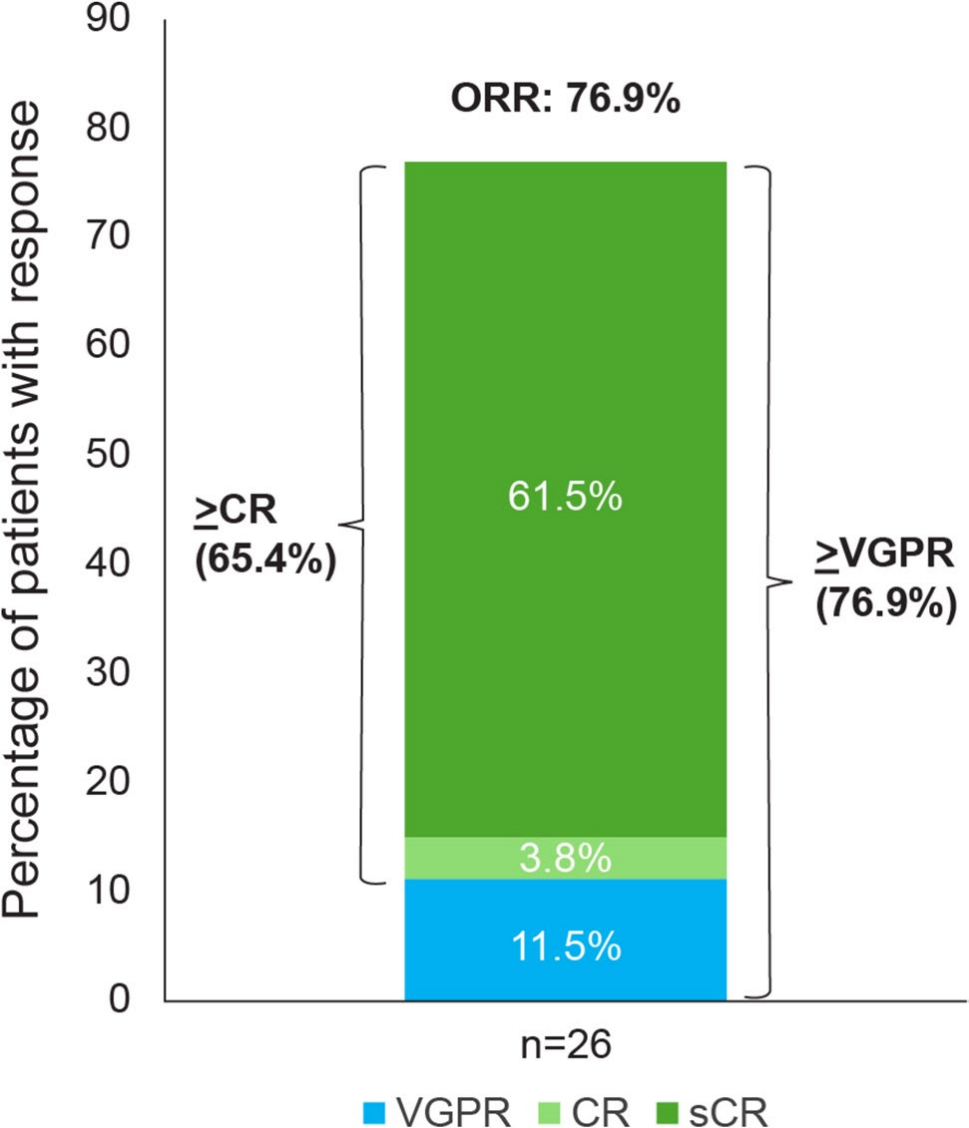
Overall response rate (phase 2). *CR* complete response, *ORR* overall response rate, *PR* partial response, *sCR* stringent complete response, *VGPR* very good partial response

**Fig. 2:**
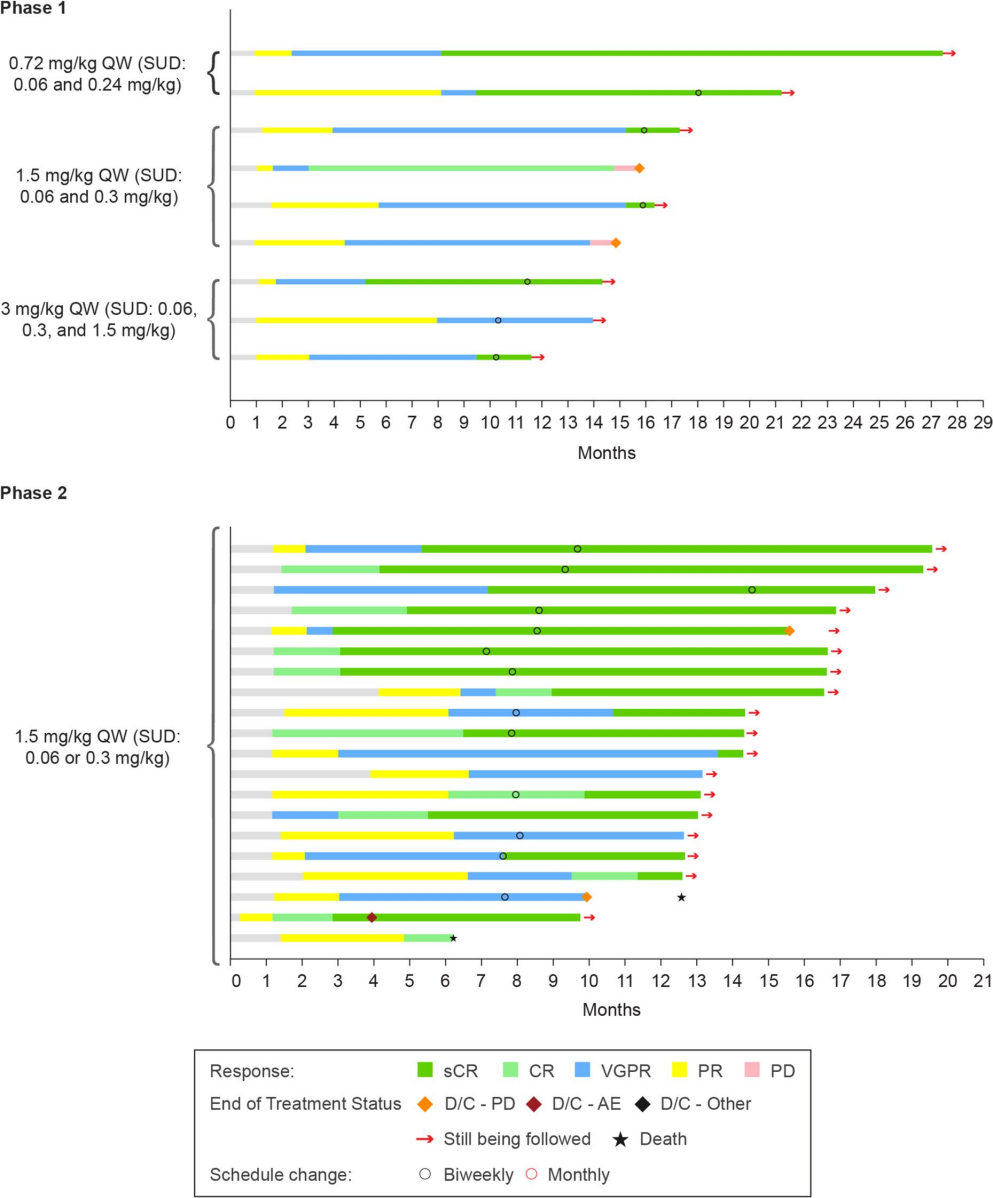
Treatment response in the patients (phases 1 and 2). *AE* adverse event, *CR* complete response, *D/C*, discontinued, *PD* progressive disease, *PR* partial response, *sCR* stringent response, *SUD* step-up dose(s), *VGPR* very good partial response

In phase 2, ORR (≥ PR) was 76.9% (20/26 patients; 95% CI 56.4–91.0); 20 (76.9%; [95% CI 56.4–91.0]) patients achieved ≥ VGPR and 17 (65.4%; [95% CI 44.3–82.8]) patients achieved ≥ CR (Fig. 1, Table 3). Of the 26 patients, 18 patients met criteria for Q2W switch and 13 switched from QW to Q2W dose (Fig. 2). At 12 months 89.7% of responders were event-free, with OS rate of 76%, and PFS rate of 75.2%. Median DOR, median PFS, and median OS were not reached with a median follow-up for 14.32 months (Supplementary Fig. 3). The overall MRD negativity rate (10^–5^) was 38.5% (95% CI 20.2–59.4) (Supplementary Table 3). In 12 patients with evaluable MRD sample, the MRD negativity rate (10^–5^) was 83.3% (95% CI 51.6–97.9).

**Table 3:** Summary of efficacy results (phase 2; all treated analysis set)

Parameters	Phase 2	
	n (%)	95% CI
sCR	16 (61.5)	40.6–79.8
CR	1 (3.8)	0.1–19.6
VGPR	3 (11.5)	2.4–30.2
PR	0	(NE, NE)
SD	3 (11.5)	2.4–30.2
Progressive disease	1 (3.8)	0.1–19.6
NE	2 (7.7)	0.9–25.1
Overall response (sCR + CR + VGPR + PR)^a^	20 (76.9)	56.4–91.0
VGPR or better (sCR + CR + VGPR)	20 (76.9)	56.4–91.0
CR or better (sCR + CR)	17 (65.4)	44.3–82.8
MRD negativity rate (10^−5^)	10 (38.5)	20.2–59.4
Time to response, median (range), months		
Time to first response (≥ PR)	1.22 (0.2–4.1)	
Time to best response	5.88 (2.9–13.6)	
Time to ≥ VGPR	2.12 (1.2–6.7)	
Time to ≥ CR	4.86 (1.2–13.6)	

*CI* confidence interval, *CR* complete response, *MRD* minimum residual response, *NE* not evaluable, *ORR* overall response rate, *PR* partial response, *sCR* stringent CR, *SD* stable disease, *VGPR* very good partial response

Responses were assessed by computerized algorithm

^a^ORR significantly exceeded the null hypothesis rate of < 20% based on a one-sided exact binominal test with a significance level of 0.025 (p < .0001)

### Pharmacokinetics

Fourteen patients in phase 1 and 26 patients in phase-2 were included in the PK analysis set. In phase 1, the mean C_max_ and AUC at Cycle 1 Day 1 and Cycle 3 Day 1 had increased proportionally per dose level (Supplementary Table 6). In phase 2, the mean serum trough concentration (C_trough_) values of teclistamab were comparable with the concentrations from cohort 2 (RP2D cohort) of phase 1. The mean (C_trough_) was maintained at or above the maximum value of 90% maximal effective concentration (EC_90_) identified in an ex vivo cytotoxicity assay. 

### Pharmacodynamics

A rapid decrease in sBCMA was observed in most responders within the first month of treatment in phase 1, and a greater reduction in sBCMA occurred in patients with deeper responses treated with RP2D of teclistamab. In phase 2, a rapid decrease in sBCMA was observed in most of the responders (≥ PR) by Cycle 2 Day 1 (14/20 [70%] patients) and continued till Cycle 4 Day 1 (17/20 [85.0%] patients).

### Immunogenicity

In phase 1, no patients were identified as positive for antibodies to teclistamab. In phase 2, 1 out of 25 anti-drug antibody-evaluable patients was identified as positive for anti-teclistamab antibodies at Cycle 3 Day 1 with low titer; but was identified as negative at the subsequent visit, Cycle 4 Day 1.

## Discussion

This is the first dedicated phase-1/2 study of teclistamab, a BCMA-targeted bispecific antibody in Japanese patients with RRMM and with an extensive history of prior treatment. Teclistamab demonstrated clinically meaningful responses that were durable and deepened over time, with a manageable safety profile in heavily pretreated patients with triple-class-exposed RRMM.

The majority of patients in the current study who switched to a Q2W dose schedule (post a PR or better for ≥ 6-months) maintained their response at the clinical cutoff date. The flexibility of the Q2W schedule could potentially reduce the burden of administration on both patients and healthcare providers. The safety of teclistamab was assessed at 3 dose levels, at QW dose, during phase 1 (0.72 mg/kg, 1.5 mg/kg, and 3 mg/kg). The safety profile was consistent across cohorts, with no dose-dependent trend and no DLTs observed during dose-escalation for all three doses, including the RP2D in Japanese patients. The overall safety profile was consistent with observations in the global MajesTEC-1 population and aligned with the mode of action concerning T-cell activation and targeting of B-cells [20, 21]. Most common and expected TEAEs when T-cells are activated against myeloma cells regardless of causality include CRS, neurotoxic events, low blood counts, and infections that were reported in this study [22]. The CRS and neurotoxic TEAEs currently reported were of low grade (grade ≤ 2), resolved by the clinical cutoff date and none led to dose reduction or treatment discontinuation. No ICANS were reported during this study. The most common hematologic TEAEs observed in this study were neutropenia, lymphopenia, and anemia; this was comparable with the global MajesTEC-1 study [12, 20, 21]. To mitigate the risk of severe CRS, a SUD schedule was employed in all cohorts during phases 1 and 2 [23], successfully reducing the incidence and severity of CRS in this study. As a result, CRS events occurred early in the treatment course, with most events limited to the SUDs and first treatment doses. These events reduced with subsequent doses and resolved by the cutoff date. BCMA-targeted T-cell redirecting therapies have also been associated with an increased susceptibility to infectious complications due to their mechanism of action [24]. In this study although the incidence of infections of any grade (76.9%) were high, the rate of grade ≥ 3 infections were low (19.2%). Furthermore, grade ≥ 3 neutropenia events were high (73.1%) but were well managed using supportive care and neither infection or neutropenia led to any discontinuation or dose reductions. Thus, appropriate screening, prophylaxis, and management of infections, hypogammaglobulinemia, and neutropenia are recommended when treating patients with anti-BCMA bispecific antibodies [25]. One patient discontinued the study treatment due to grade 3 hyponatremia TEAE (1[3.8%]). Two grade 5 TEAEs leading to death were reported and neither of these TEAEs were considered related to the study treatment. Taken together teclistamab demonstrated a manageable safety profile in Japanese patients with no new safety concerns reported. The safety findings were consistent with the pivotal global MajesTEC-1 study assessing teclistamab monotherapy, despite variations in the demographics.

At RP2D in phase 2, responses occurred in 76.9% patients, comparable with the global MajesTEC-1 study with a majority achieving deep responses and having a VGPR (76.9%) or sCR (61.5%). In the current study, all responses deepened over time, from an initial response of PR to the best response of VGPR, CR, and sCR. Overall, teclistamab resulted in deep and durable responses in Japanese patients with triple-class-exposed RRMM and the responses were comparable with the global MajesTEC-1 pivotal study [12].

From the PK results it was observed that teclistamab serum C_trough_ values were comparable with the observed concentrations from the RP2D cohort in phase 1. The serum concentration of teclistamab at RP2D was sustained over the predetermined target level based on an experimentally determined maximum value range of EC_90_. No patients in phase 1, and 1 patient in phase 2 were anti-teclistamab antibody positive. In the current study a rapid decrease in sBCMA was observed in most responders within the first month of treatment in the patients treated with RP2D in phase-1 and a similar trend was observed in phase 2. A rapid decrease in sBCMA levels in responders is an important indicator of treatment efficacy and improved clinical outcomes including longer progression-free survival in patients with RRMM [26]. Taken together the PK, PD, and immunogenicity results were in line with the study assessing teclistamab in the global MajesTEC-1 non-Japanese population [12]. Limitations include the open-label and single-arm study design with a lack of a comparison arm and the survival data are not mature in this ongoing study.

In conclusion, SC teclistamab at the QW RP2D of 1.5 mg/kg (with 50% patients switching to Q2W dose, post ≥ 6-months of PR or better), established in the pivotal MajesTEC-1 global study, demonstrated manageable safety in triple-class-exposed Japanese patients with RRMM. Teclistamab resulted in deep and durable responses in heavily pretreated patients with RRMM. Overall teclistamab demonstrated favorable results as monotherapy in Japanese patients with heavily pretreated RRMM, supporting the potential for a new SOC for patients with RRMM in Japan.

## Supplementary Information

Below is the link to the electronic supplementary material.

Supplementary file1 (DOCX 403 KB)

## Data Availability

The data sharing policy of Janssen Pharmaceutical Companies of Johnson & Johnson is available at https://www.janssen.com/clinical-trials/transparency. As noted on this site, requests for access to the study data can be submitted through Yale Open Data Access [YODA] Project site at http://yoda.yale.edu.
