# Supplementary material for: A Phase 1/2 study of teclistamab, a humanized BCMA × CD3 bispecific Ab in Japanese patients with relapsed/refractory MM
Source: Int J Hematol. 2024 Nov 28;121(2):222–31. doi: 10.1007/s12185-024-03884-z (PMC11782335; doi:10.1007/s12185-024-03884-z)
Supplement: Supplementary file 1 — Supplementary file1 (DOCX 403 KB) [file 12185_2024_3884_MOESM1_ESM.docx]

# **Supplemental Information**

**Methods**

**Pharmacokinetics, pharmacodynamics, and immunogenicity analysis**

The blood samples were collected during the first 4 cycles of the treatment. For pharmacokinetic analysis, samples were collected on Days 1, 2, 3, 4, 6, 8, and 15 of Cycle 1; Days 1 and 15 of Cycle 2; Days 1, 2, 3, 4, 6, 8, and 15 of Cycle 3; Day 1 of Cycle 4 and subsequent cycles. For pharmacodynamic analysis, samples were collected prior to SUD 1, on Day 4 of Cycle 1, and Day 4 of Cycle 3. For immunogenicity analysis, prior to SUD 1, on Day 1, 8, 15 of Cycle 1, Day 1 of Cycle 2, Cycle 3, Cycle 4, and subsequent cycles.

**Supplementary Table 1:** Dose-limiting toxicity criteria^a^

| **Non-hematological toxicity** | |
| --- | --- |
| TLS^a,b^ | Grade ≥3 with clinical sequelae that does not resolve within 72 h |
| Other non-hematological toxicity^b^ | - Grade ≥3 sARR or CRS that does not resolve to grade ≤1 within 48 h - Grade ≥3, except: - Grade 3 asthenia, fever, or constipation - Grade 3 nausea, vomiting, diarrhea, or pain that resolve within 72 h |
| **Clinical laboratory abnormalities** |  |
| AST or ALT^b^ | - Grade 3 unless resolved to grade ≤1 (or baseline) within 72 hours - Grade 4 - Meeting criteria for Hy’s law^c^ |
| Lipase or amylase^b^ | Grade ≥3 associated with clinical or radiological evidence of pancreatitis |
| Other chemistry abnormalities^b^ | Grade ≥3 and associated with clinical complications unless resolved to grade ≤1 (or baseline) within 72 h |
| **Hematological toxicity** | |
| Neutrophil count decreased^b^ | Grade 4 for >7 days^d^ |
| Platelet count decreased^b^ | - Grade 3 with clinically significant bleeding or the requirement for platelet transfusion (prophylactic transfusions excluded) - Grade ≥4 |

^a^The SET will evaluate all TLS events and decide if the circumstances meet the criteria for DLT. ^b^If the AE is part of a syndrome (eg, TLS, CRS, or sARR), then the grade of the syndrome should dictate the DLT assessment. ^c^Hy’s Law criteria, defined as ALT or AST value ≥3×ULN, total bilirubin ≥2×ULN, and alkaline phosphatase ≤2×ULN; with no alternative etiology. ^d^More frequent laboratory monitoring including CBC and differential should be initiated to document the start and the resolution of the specific toxicity; persistence needs to be documented by a CBC with differential blood count immediately after the time limitation for the specific toxicity has expired.

AE, adverse event; ALT, alanine aminotransferase; AST, aspartate aminotransferase; CBC, complete blood count; CRS, cytokine release syndrome; DLT, dose-limiting toxicity; sARR, systemic administration-related reactions; SET, study evaluation team; TLS, tumor lysis syndrome; ULN, upper limit of normal

**Supplementary Table 2:** Safety summary

| **Characteristics, n (%)** | **Phase 1^a^** | | | | **Phase 2^b^** |
| --- | --- | --- | --- | --- | --- |
|  | Cohort 1  0.72 mg/kg QW  (n=5) | Cohort 2  1.5 mg/kg QW  (n=5) | Cohort 3  3 mg/kg QW  (n=4) | Total  (n=14) | RP2D  1.5 mg/kg QW  (n=26) |
| Patients with ≥1 TEAE  (any grade) | 4 (80.0) | 5 (100.0) | 4 (100.0) | 13 (92.9) | 26 (100.0) |
| Treatment-related | 4 (80.0) | 5 (100.0) | 4 (100.0) | 13 (92.9) | 25 (96.2) |
| Patients with ≥1 TEAEs  (grade ≥3) | 4 (80.0) | 5 (100.0) | 4 (100.0) | 13 (92.9) | 23 (88.5) |
| Serious TEAEs | 2 (40.0) | 2 (40.0) | 2 (50.0) | 6 (42.9) | 13 (50.0) |
| Treatment-related | 2 (40.0) | 1 (20.0) | 1 (25.0) | 4 (28.6) | 6 (23.1) |
| Patients with ≥1 TEAEs leading to discontinuation | 0 | 0 | 0 | 0 | 1 (3.8) |
| Serious TEAEs of COVID-19 | 1 (20.0) | 0 | 0 | 1 (7.1) | 0 |
| Deaths | 0 | 0 | 0 | 0 | 7 (26.9) |
| TEAEs lead to death | 0 | 0 | 0 | 0 | 2 (7.7) |

^a^Clinical data cutoff date for phase 1 is September 2023. ^b^Clinical data cutoff date for phase 2 is April 2024.

COVID-19, corona virus disease-19; RP2D, recommended phase-2 dose; TEAE, treatment-emergent adverse event; QW, once weekly.

**Supplementary Table 3:** Cytokine release syndrome: characteristics and treatment

| **Parameter** | **Phase 1** | | | | **Phase 2** |
| --- | --- | --- | --- | --- | --- |
|  | Cohort 1  0.72 mg/kg QW  (n=5) | Cohort 2  1.5 mg/kg QW  (n=5) | Cohort 3  3 mg/kg QW  (n=4) | Total  (n=14) | RP2D  1.5 mg/kg QW  (n=26) |
| Patients with CRS | 2 (40.0) | 3 (60.0) | 3 (75.0) | 8 (57.1) | 21 (80.8) |
| Grade 1 | 2 (40.0) | 3 (60.0) | 3 (75.0) | 8 (57.1) | 18 (69.2) |
| Grade 2 | 0 | 0 | 0 | 0 | 3 (11.5) |
| Patients with multiple CRS events | 1 (20.0) | 3 (60.0) | 1 (25.0) | 5 (35.7) | 5 (19.2) |
| CRS event |  |  |  |  |  |
| Pyrexia | 2 (40.0) | 3 (60.0) | 3 (75.0) | 8 (57.1) | 20 (76.9) |
| Chills | 1 (20.0) | 0 | 0 | 1 (7.1) | 0 |
| Headache | 0 | 1 (20.0) | 1 (25.0) | 2 (14.3) | 1 (3.8) |
| Vomiting | 0 | 1 (20.0) | 0 | 1 (7.1) | 0 |
| Increased alanine  aminotransferase | 0 | 1 (20.0) | 0 | 1 (7.1) | 0 |
| Increased aspartate  aminotransferase | 0 | 1 (20.0) | 0 | 1 (7.1) | 0 |
| Back pain | 0 | 0 | 1 (25.0) | 1 (7.1) | 0 |
| Disseminated intravascular coagulation | 0 | 0 | 0 | 0 | 1 (3.8) |
| Fluid retention | 0 | 0 | 0 | 0 | 1 (3.8) |
| Hypoxia | 0 | 0 | 0 | 0 | 1 (3.8) |
| Hypotension | 0 | 0 | 0 | 0 | 1 (3.8) |
| Events lead to death | 0 | 0 | 0 | 0 | 0 |
| Occurrence of CRS^a^ | | | | | |
| SUD 1 | 1 (20.0) | 2 (40.0) | 2 (50.0) | 5 (35.7) | 9 (34.6) |
| SUD 2 | 2 (40.0) | 2 (40.0) | 0 | 4 (28.6) | 9 (34.6) |
| SUD 3 | - | - | 1 (25.0) | 1 (7.1) | - |
| Repeat SUD^b^ | 0 | 0 | 0 | 0 | 1 (3.8) |
| Cycle 1 Day 1 | 1 (20.0) | 1 (20.0) | 0 | 2 (14.3) | 4 (15.4) |
| Cycle 1 Day 8 | 0 | 2 (40.0) | 1 (25.0) | 3 (21.4) | 1 (3.8) |
| Cycle 1 Day 15 | 0 | 0 | 0 | 0 | 1 (3.8) |
| Cycle 1 Day 22 | - | - | - | - | 0 |
| Cycle 2+ | 0 | 0 | 0 | 0 | 2 (7.7) |
| Time to onset, median (range), days | 2 .0 (2.0–2.0) | 2.0 (1.0–7.0) | 2.0 (2.0–2.0) | 2.0 (1.0–7.0) | 2.0 (1.0–5.0) |
| Duration, median (range), days | 3.5 (2.0–4.0) | 3.0 (1.0–10.0) | 2.5 (2.0–3.0) | 3.0 (1.0–10.0) | 3.0 (1.0–24.0) |
| Received supportive measures for CRS, n (%) | | | | | |
| Tocilizumab | 1 (20.0) | 3 (60.0) | 3 (75.0) | 7 (50.0) | 17 (65.4) |
| Acetaminophen | 2 (40.0) | 2 (40.0) | 2 (50.0) | 6 (42.9) | 17 (65.4) |
| IV fluids | 1 (20.0) | 1 (20.0) | 1 (25.0) | 3 (21.4) | 3 (11.5) |
| Corticosteroids | 0 | 0 | 0 | 0 | 3 (11.5) |
| Oxygen used | 0 | 0 | 0 | 0 | 1 (3.8) |
| Other | 0 | 1 (20.0) | 0 | 1 (7.1) | 2 (7.7) |

All data were presented in n (%) unless specified. ^a^Patients may appear in more than one category. Occurrence is based on the last treatment visit on or prior to the day in which the TEAE occurred. ^b^Prior to Cycle 1.

CRS, cytokine release syndrome; IV, intravenous; RP2D, recommended Phase-2 dose; SUD, step-up dose; QW, once weekly.

**Supplementary Table 4:** Neurotoxic events: characteristics and treatment

| **Parameter** | **Phase 1** | | | | **Phase 2** |
| --- | --- | --- | --- | --- | --- |
|  | Cohort 1  0.72 mg/kg QW  (n=5) | Cohort 2  1.5 mg/kg QW  (n=5) | Cohort 3  3 mg/kg QW  (n=4) | Total  (n=14) | RP2D  1.5 mg/kg QW  (n=26) |
| Patients with ≥1 neurotoxic event | 2 (40.0) | 1 (20.0) | 0 | 3 (21.4) | 2 (7.7) |
| Grade 1 | 1 (20.0) | 1 (20.0) | 0 | 2 (14.3) | 2 (7.7) |
| Grade 2 | 1 (20.0) | 0 | 0 | 1 (7.1) | 0 |
| Neurotoxic event |  |  |  |  |  |
| Headache | 2 (40.0) | 0 | 0 | 2 (14.3) | 1 (3.8) |
| Peripheral sensory neuropathy | 0 | 1 (20.0) | 0 | 1 (7.1) | 0 |
| Somnolence | 0 | 0 | 0 | 0 | 1 (3.8) |
| ICANS | 0 | 0 | 0 | 0 | 0 |
| Events lead to death | 0 | 0 | 0 | 0 | 0 |
| Occurrence of neurotoxcity^a^ |  |  |  |  |  |
| SUD 1 | 0 | 0 | 0 | 0 | 1 (3.8) |
| SUD 2 | 1 (20.0) | 0 | 0 | 1 (7.1) | 0 |
| SUD 3 | - | - | 0 | 0 | 0 |
| Repeat SUD^b^ | 0 | 0 | 0 | 0 | 0 |
| Cycle 1 Day 1 | 1 (20.0) | 0 | 0 | 1 (7.1) | 0 |
| Cycle 1 Day 8 | 0 | 0 | 0 | 0 | 0 |
| Cycle 1 Day 15 | 0 | 0 | 0 | 0 | 0 |
| Cycle 2+ | 0 | 1 (20.0) | 0 | 1 (7.1) | 1 (3.8) |
| Time to onset, median (range), days | 2.5 (2.0– 3.0) | 8.0 (8.0– 8.0) | - | 3.0 (2.0–8.0) | 33.0 (2.0–64.0) |
| Duration, median (range), days | 9.5  (3.0– 16.0) | 310.0 (310.0– 310.0 | - | 16.0 (3.0–310.0) | 3.0 (2.0–4.0) |
| Received supportive measures, n (%)^c^ | | | | | |
| Dexamethasone | 0 | 1 (20.0) | 0 | 1 (7.1) | 0 |
| Other | 2 (40.0) | 1 (20.0) | 0 | 3 (21.4) | 0 |

All data were presented in n (%) unless specified. ^a^Patients may appear in more than one category. Occurrence is based on the last treatment visit on or prior to the day in which the TEAE occurred. ^b^Prior to Cycle 1. ^c^Supportive measures to treat only neurotoxicity and symptoms of ICANS were included.

ICANS, immune effector cell-associated neurotoxicity syndrome; RP2D, recommended phase-2 dose; SUD, step-up dose; QW, once weekly.

**Supplementary Table 5:** Treatment emergent infections

| **Parameter** | **Phase 1** | | | | **Phase 2** |
| --- | --- | --- | --- | --- | --- |
|  | Cohort 1  0.72 mg/kg QW  (n=5) | Cohort 2  1.5 mg/kg QW  (n=5) | Cohort 3  3 mg/kg QW  (n=4) | Total  (n=14) | RP2D  1.5 mg/kg QW  (n=26) |
| Patients with ≥1 infections |  |  |  |  |  |
| Any grade | 4 (80.0) | 3 (60.0) | 2 (50.0) | 9 (64.3) | 20 (76.9) |
| Grade 3 or 4 | 1 (20.0) | 0 | 1 (25.0) | 2 (14.3) | 5 (19.2) |
| Event (any grade) | | | | | |
| Nasopharyngitis | 1 (20.0) | 1 (20.0) | 0 | 2 (14.3) | 8 (30.8) |
| Tinea pedis | 0 | 2 (40.0) | 0 | 2 (14.3) | 0 |
| COVID-19 | 1 (20.0) | 0 | 0 | 1 (7.1) | 1 (3.8) |
| COVID-19 pneumonia | 1 (20.0) | 0 | 0 | 1 (7.1) | 0 |
| Cystitis | 1 (20.0) | 0 | 0 | 1 (7.1) | 2 (7.7) |
| Oral candidiasis | 1 (20.0) | 0 | 0 | 1 (7.1) | 0 |
| Pharyngitis | 0 | 1 (20.0) | 0 | 1 (7.1) | 0 |
| Sepsis | 0 | 0 | 1 (25.0) | 1 (7.1) | 1 (3.8) |
| Skin infection | 1 (20.0) | 0 | 0 | 1 (7.1) | 0 |
| Subcutaneous abscess | 0 | 0 | 1 (25.0) | 1 (7.1) | 0 |
| Tinea infection | 1 (20.0) | 0 | 0 | 1 (7.1) | 0 |
| Upper respiratory tract  infection | 0 | 0 | 1 (25.0) | 1 (7.1) | 2 (7.7) |
| Cytomegalovirus infection | 0 | 0 | 0 | 0 | 3 (11.5) |
| Bronchitis | 0 | 0 | 0 | 0 | 2 (7.7) |
| Influenza | 0 | 0 | 0 | 0 | 2 (7.7) |
| Pneumonia | 0 | 0 | 0 | 0 | 2 (7.7) |
| Sinusitis | 0 | 0 | 0 | 0 | 2 (7.7) |
| Acute sinusitis | 0 | 0 | 0 | 0 | 1 (3.8) |
| Bacteremia | 0 | 0 | 0 | 0 | 1 (3.8) |
| Bacterial infection | 0 | 0 | 0 | 0 | 1 (3.8) |
| Bronchopulmonary aspergillosis | 0 | 0 | 0 | 0 | 1 (3.8) |
| Cellulitis | 0 | 0 | 0 | 0 | 1 (3.8) |
| Chronic sinusitis | 0 | 0 | 0 | 0 | 1 (3.8) |
| Cytomegalovirus chorioretinitis | 0 | 0 | 0 | 0 | 1 (3.8) |
| Folliculitis | 0 | 0 | 0 | 0 | 1 (3.8) |
| Gastroenteritis | 0 | 0 | 0 | 0 | 1 (3.8) |
| Oral herpes | 0 | 0 | 0 | 0 | 1 (3.8) |
| Periodontitis | 0 | 0 | 0 | 0 | 1 (3.8) |
| Pneumonia cytomegaloviral | 0 | 0 | 0 | 0 | 1 (3.8) |
| Sialoadenitis | 0 | 0 | 0 | 0 | 1 (3.8) |

COVID, coronavirus disease; RP2D, recommended phase-2 dose; QW, once weekly.

**Supplementary Table 6:** Pharmacokinetic results of phase-1 and 2

| **Parameters** | **Phase 1** | | | **Phase 2** |
| --- | --- | --- | --- | --- |
|  | Cohort 1  0.72 mg/kg QW  (n=5) | Cohort 2  1.5 mg/kg QW  (n=5) | Cohort 3  3 mg/kg QW  (n=4) | RP2D  1.5 mg/kg QW  (n=26) |
| Cycle 1 Day 1 |  |  |  |  |
| n | 5 | 5 | 4 | 24 |
| C_max_, μg/mL | 4.61 (4.64) | 6.96 (3.13) | 19.4 (6.53) | 1.15 (0.41) |
| T_max_, median (range), h | 99.28  (67.57–167.13) | 142.50  (69.55–191.63) | 96.80  (24.00–192.05) | - |
| AUC_last_, μg·h/mL | 586 (656) | 907 (524) | 2577 (772) | - |
| T_last,_ h | 167.13  (165.38–195.97) | 167.50  (142.50–191.63) | 167.68  (143.97–192.05) | - |
| Cycle 3 Day 1 |  |  |  |  |
| n | 3 | 4 | 3 | 22 |
| C_max_, μg/mL | - | 19.1 (6.01) | 39.0 (10.8) | 18.26 (6.48) |
| C_trough_, μg/mL | 14.6 (9.29) | 13.1 (3.22) | 28.8 (4.20) | - |
| T_max_, median (range), h | - | 46.32  (23.05–68.30) | 46.62  (23.30–48.20) | - |
| AUC_last_, μg·h/mL | - | 2792 (665) | 6516 (2193) | - |
|  |  |  |  |  |
| T_last,_ h | - | 166.42  (165.73–169.47) | 189.03  (166.00–214.73) | - |
| CL/F, L/h | - | 0.0267 (0.00313) | 0.0257 (0.0141) | - |
| All values are expressed in terms of mean (SD) unless specified. AUC_last_, area under the curve from the time of dosing to the last measurable concentration; C_max_, maximum serum concentration; C_trough_, serum concentration just prior to next administration of drug.  CL/F, apparent clearance; PK, pharmacokinetics; QW, once weekly; QD, once daily; SD, standard deviation; t_1/2_, half-life; T_max_, time to maximum concentration; T_last_, time of the last sample collected | | | | |

**Supplementary Fig. 1:** Study overview


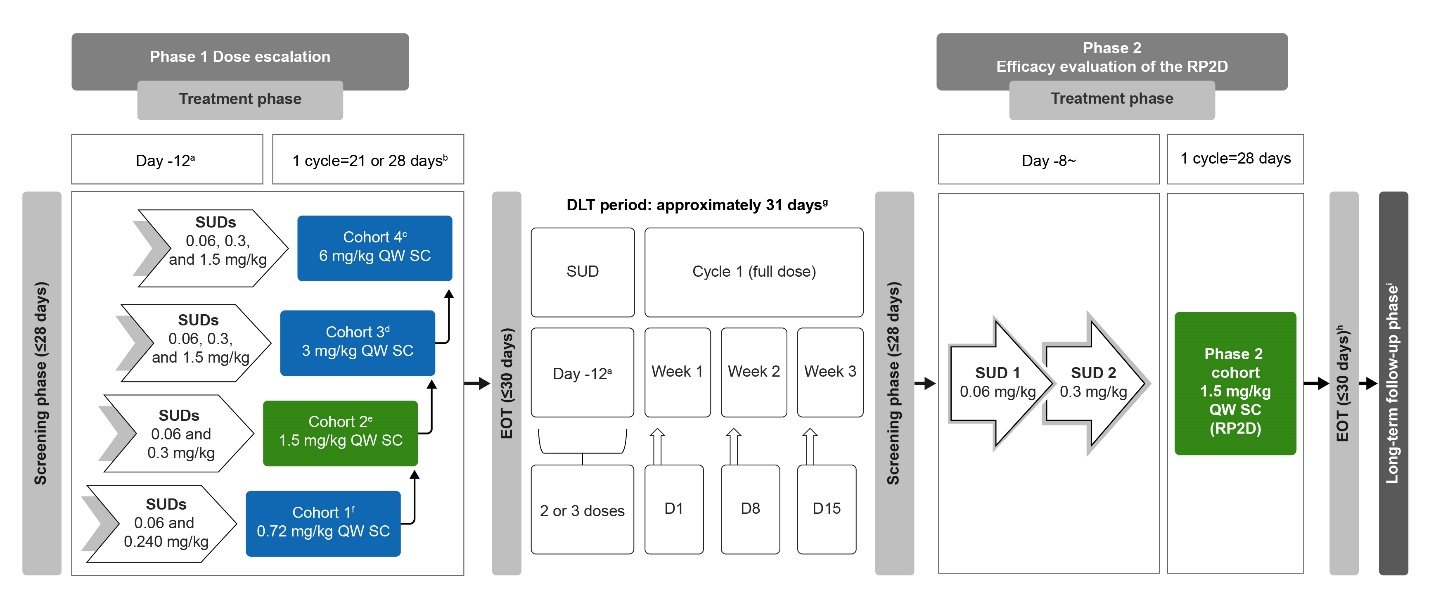
^a^For 0.72 mg/kg and 1.5 mg/kg cohorts, 2 SUD start from 4 to 8 days prior to the first treatment dose. For

3 mg/kg and 6 mg/kg cohorts, 3 SUD start from 6 to 12 days prior to the first treatment dose. ^b^28 days cycle is applied to 6 mg/kg biweekly and monthly dose schedule. ^c^6 mg/kg weekly (cycle 1 and 2), biweekly (cycle 3 to 6), monthly (beginning at cycle 7) (SC): two doses level higher than the RP2D of 64007957MMY1001 study. ^d^3 mg/kg weekly (SC): one dose level higher than the RP2D of 64007957MMY1001 study. ^e^1.5 μg/kg weekly (SC): same dose as RP2D of 64007957MMY1001 study. ^f^SUD schedule consists of 2 doses for 0.72 mg/kg weekly and 1.5 mg/kg weekly cohort and 3 doses for 3 mg/kg weekly and 6 mg/kg cohorts. ^g^For 0.72 mg/kg and 1.5 mg/kg cohorts, DLT period is approximately 28 days. For 3 mg/kg and 6 mg/kg cohorts, DLT period is approximately 31 days.

^h^Long follow-up continues until death, withdrawal of consent for study participation, or lost to follow-up.

D, day; DLT, dose-limiting toxicity; EOT, end-of-treatment; PD, progressive disease; RP2D, recommended phase-2 dose; SC, subcutaneous; SUD, step-up dose.

**Supplementary Fig. 2:** Overall response rate (phase 1)


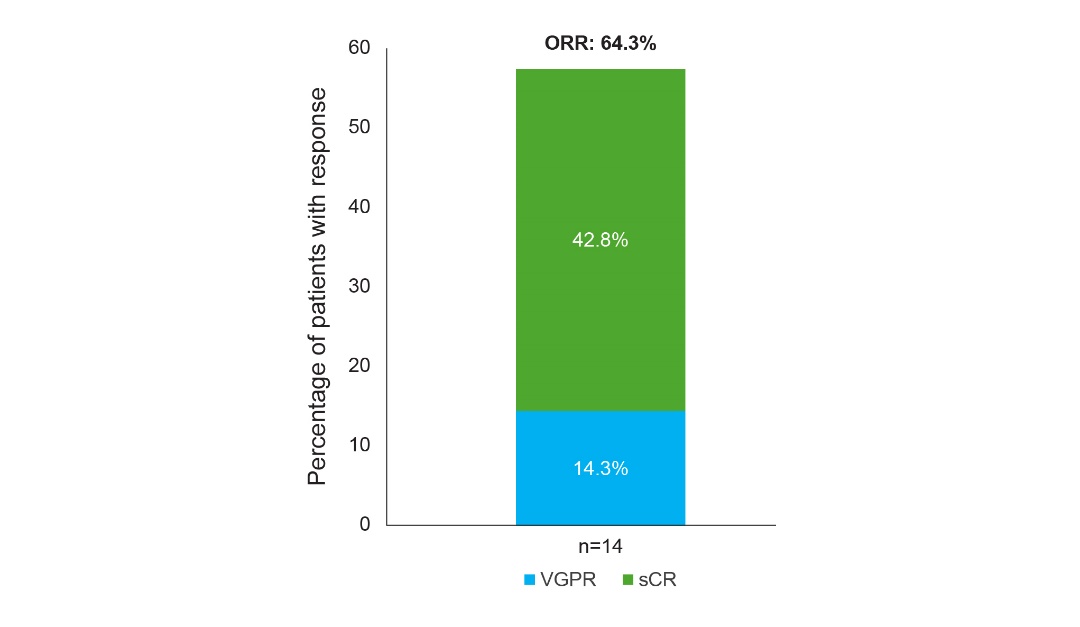


ORR, overall response rate; sCR, stringent complete response; VGPR, very good partial response

**Supplementary Fig. 3:** Kaplan-Meier plots (phase 2)

1.
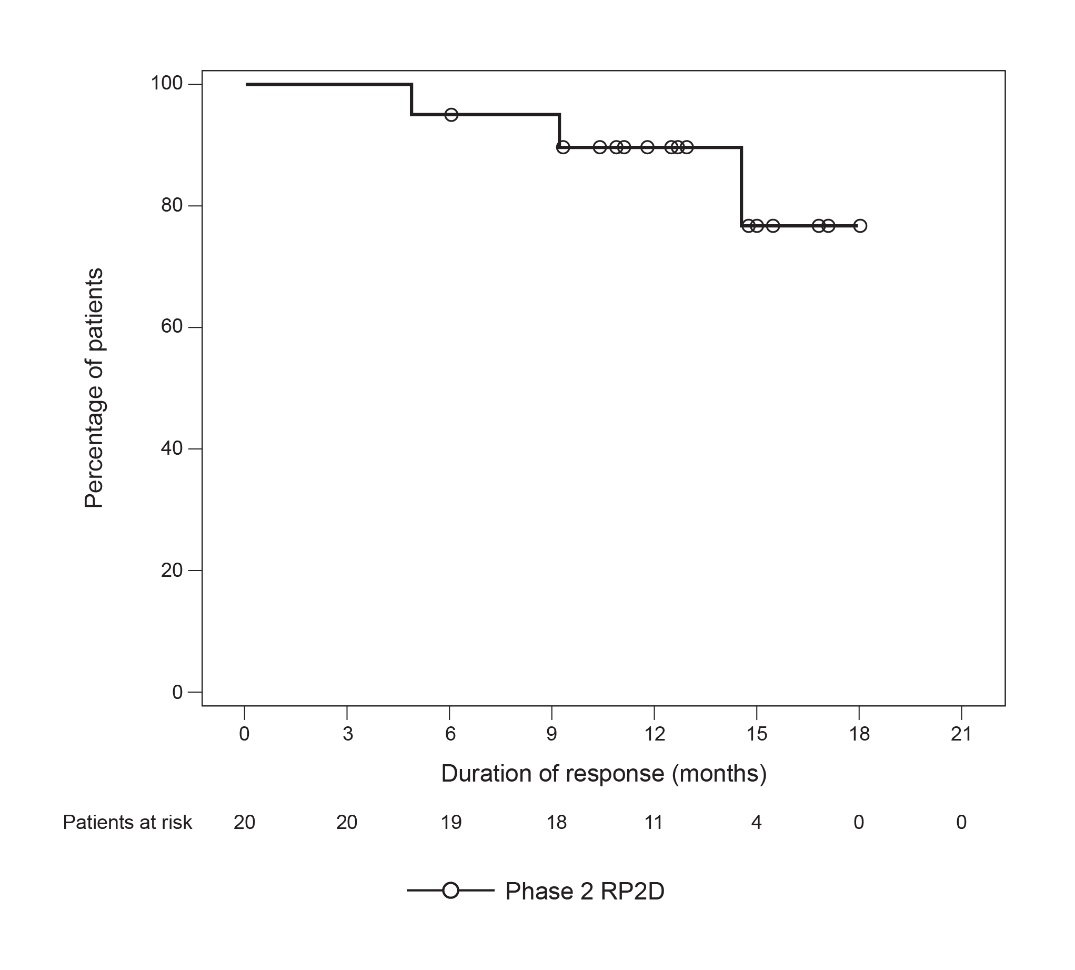
Duration of response
2.
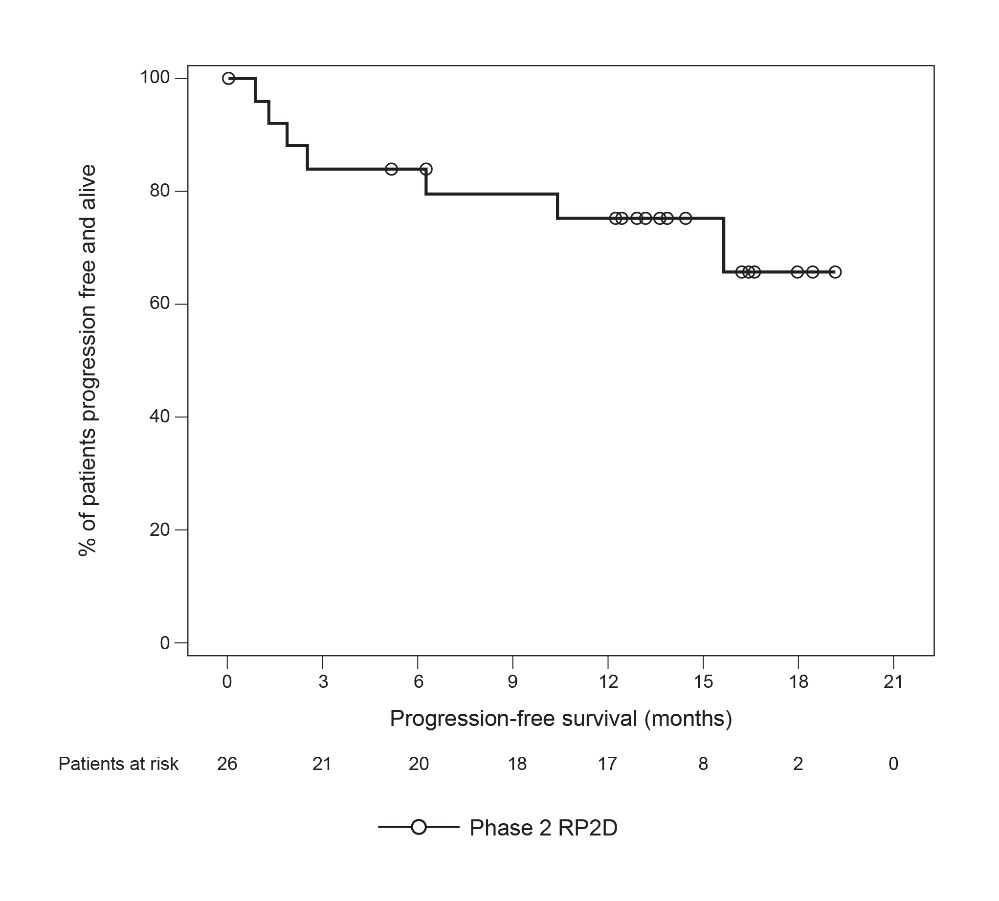
Progression-free survival
3.
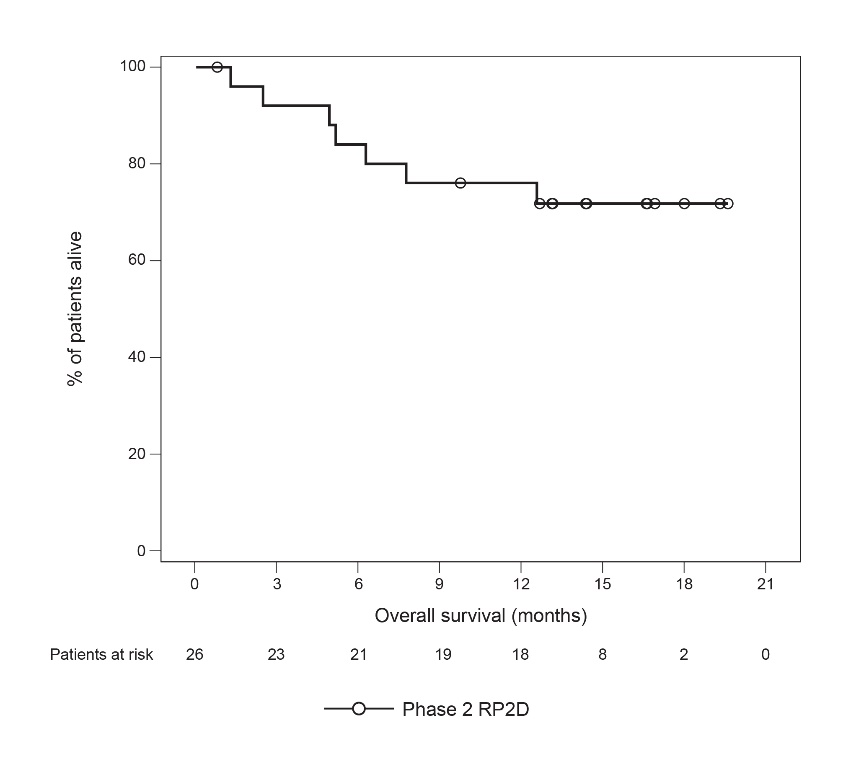
Overall survival

RP2D, recommended phase-2 dose
